# Improved survival outcomes with anakinra over etoposide-based therapies for the management of adults with hemophagocytic lymphohistiocytosis: a retrospective multicenter research network study

**DOI:** 10.1177/20406207241245517

**Published:** 2024-04-16

**Authors:** Benjamin J. Lee

**Affiliations:** Department of Pharmacy, Chao Family Comprehensive Cancer Center, University of California Irvine Health, 101 The City Drive South, Building 23, Room 275, Orange, CA 92868, USA; Department of Clinical Pharmacy Practice, School of Pharmacy and Pharmaceutical Sciences, University of California, Irvine, Irvine, CA, USA

**Keywords:** anakinra, etoposide, hemophagocytic lymphohistiocytosis, ruxolitinib

## Abstract

**Background:**

Hemophagocytic lymphohistiocytosis (HLH) is a rare life-threatening, hyperinflammatory syndrome for which etoposide-based regimens have historically been the standard of care. Recent reports have described positive outcomes with the utilization of ruxolitinib or anakinra although these studies are often limited to small samples.

**Objectives:**

We aimed to compare the efficacy of ruxolitinib, anakinra, and etoposide-based therapies for the management of HLH in adult patients.

**Design:**

We performed a population-based, multicenter, retrospective cohort study utilizing the TriNetX Networks database.

**Methods:**

Adult patients (⩾18 years) diagnosed with HLH who received first-line treatment with ruxolitinib, anakinra, or etoposide between 2008 and 2023 were analyzed. The primary endpoint was overall survival (OS) at 1 year. A 1:1 propensity-score matching analysis was utilized.

**Results:**

Anakinra (*p* = 0.020) but not ruxolitinib (*p* = 0.19) resulted in a significantly higher 1-year OS when compared with etoposide-based therapies.

**Conclusions:**

Anakinra is effective for the management of adult patients with HLH.

## Introduction

Hemophagocytic lymphohistiocytosis (HLH) is a rare life-threatening, hyperinflammatory syndrome that is commonly managed with an etoposide-based regimen such as the HLH-94 protocol.^1^ Although inciting factors for the development of HLH are widely heterogeneous, with the most common triggers identified as hematologic malignancy, infection, or autoimmune disease, clinical outcomes overall remain poor. Recent reports have described positive outcomes with the utilization of ruxolitinib or anakinra although these publications are limited to case reports and small studies.^2–12^ Herein, we aim to compare survival outcomes of adult patients with HLH who were treated with ruxolitinib, anakinra, and etoposide-based therapies.

## Methods

We performed a multicenter, retrospective electronic health cohort study utilizing the TriNetX platform,^13^ a global, federated health research network consisting of 114 participating healthcare organizations (HCOs) at the time of writing. The TriNetX platform provides access to real-time, real-world electronic health records in an aggregate and de-identified form that aligns with the Health Insurance Portability and Accountability Act, the US Federal Law, and the EU Data Protection Law Regulation 2016/679. Accessible data for research purposes include the International Classification of Diseases, Tenth Revision (ICD-10) diagnosis codes, as well as demographics, laboratory data elements, procedural data, and medication administrations. To protect the identity of patients included in the TriNetX database, all queries resulting in a total patient count of less than 10 are not reported.

We sought to assess the efficacy of ruxolitinib, anakinra, and etoposide-based therapies in the management of adults with HLH. Patients with an ICD-10 diagnosis code for HLH (D76.0) between January 2008 and December 2023 and 18–80 years of age at diagnosis were assessed for inclusion. Patients were excluded if they received a chimeric antigen receptor T-cell therapy or if they received emapalumab or tocilizumab up to 1 year after HLH diagnosis. Patients with a recent history of hematologic malignancy were defined as having a composite ICD-10 code for malignant neoplasms of lymphoid, hematopoietic, and related tissue (C81–C96), within 1 year of HLH diagnosis. The primary outcome of interest was overall survival (OS) at 1 year. To determine whether practice changes over time influenced treatment outcomes, subgroup analyses were also performed over two time periods, January 2008 to December 2015 and January 2016 to December 2023. Analyses that compared ruxolitinib with anakinra or etoposide only included those treated between January 2015 and December 2023 as ruxolitinib utilization was not found prior to 2015 in the TriNetX database. Bivariate regression analysis was performed on baseline characteristics associated with worse OS to determine variables included in the matching analysis. A comparison of 1-year OS based on identified characteristics was also performed between treatment arms. A 1:1 propensity-score matching utilizing logistic regression was performed based on age, male sex, history of hematologic malignancy, management by critical care services, and key baseline laboratory values at initial diagnosis which included: absolute neutrophil count (ANC), serum ferritin, fibrinogen, and triglycerides for the primary outcome of interest. The secondary outcome of interest was the development of secondary hematologic malignancy at 5 years among patients without a prior history of leukemia, lymphoma, myeloma, or other hematologic malignant processes. All statistical analyses were conducted utilizing the TriNetX Analytics platform.

## Results

**Figure 1.**
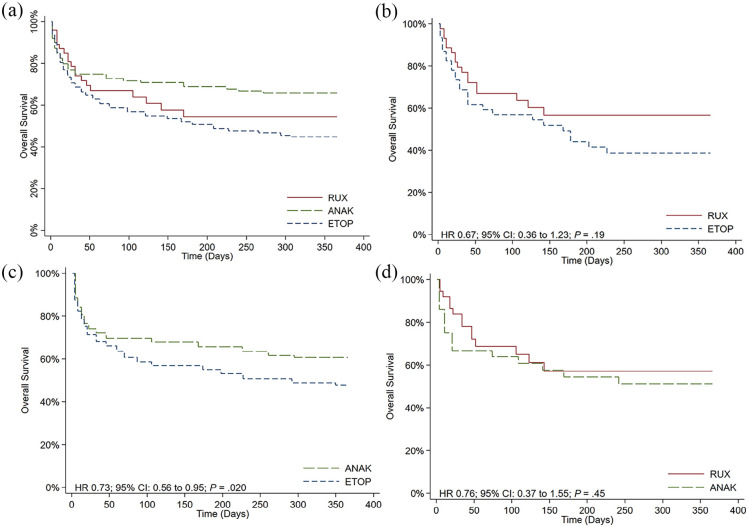
Overall survival at 1 year between (a) all patients who received RUX, ANAK, or ETOP-based regimens, (b) patients who received RUX and ETOP after the propensity-score match, (c) patients who received ANAK and ETOP after the propensity-score match, and (d) patients who received RUX and ANAK after the propensity-score match. ANAK, anakinra; ETOP, etoposide; HLH, hemophagocytic lymphohistiocytosis; RUX, ruxolitinib.

The TriNetX database was queried on 20 December 2023 and a total of 1204 patients were identified from 65 HCOs. The mean age ± SD was 48 ± 19 years and 55.9% were male. In all, 541 patients (44.9%) had a history of hematologic malignancy prior to diagnosis. Critical care services were required in 430 (35.7%) patients and the mean (±SD) serum ferritin, interleukin (IL)-2, triglyceride, and fibrinogen were 17,299 ± 35,390 mg/L, 12,035 ± 12,992 pg/mL (reported in 9.0% of patients), 306 ± 217 mg/dL, and 307 ± 195 mg/dL at baseline. The majority of patients received HLH-directed therapy with an etoposide-based regimen (*n* = 726; 60.3%) followed by anakinra (*n* = 428; 35.5%) and ruxolitinib (*n* = 48, 4.2%). OS at 1 year was 43.8% for the whole cohort and was not significantly different between ruxolitinib (54.5%) and anakinra-treated patients (65.6%; Hazard Ratio [HR] 1.27; 95% CI 0.78–2.05; *p* = 0.33) or etoposide-treated patients (44.3%; HR 0.77; 95% CI 0.48–1.22; *p* = 0.26). Patients who received an etoposide-based regimen, however, had a significantly lower 1-year OS compared to those who received anakinra (HR 1.72; 95% CI 1.41–2.11; *p* < 0.001) [Figure 1(a)]. Patient characteristics associated with worse 1-year OS included male sex (HR 1.48; 95% CI 1.22–1.80; *p* < 0.001), older age (⩾55 years old) (HR 2.82; 95% CI 2.35–3.93; *p* < 0.001), recent history of hematologic malignancy (HR 1.37; 95% CI 1.15–1.65; *p* < 0.001), ICU admission and critical care services for HLH-management (HR 2.34; 95% CI 1.95–2.81; *p* < 0.001), hyperferritinemia (⩾15,000 mg/L) (HR 1.62; 95% CI 1.36–1.92; *p* < 0.001), elevated serum triglyceride (⩾300 mg/dL) (HR 1.36; 95% CI 1.13–1.63; *p* = 0.001), hypofibrinogenemia (⩽150 mg/dL) (HR 1.25; 95% CI 1.05–1.50; *p* = 0.014), and neutropenia (ANC <1.0 × 10^9^/L) (HR 1.21; 95% CI 1.03–0.42; *p* = 0.023).

After propensity-score matching, in 46 patients who received ruxolitinib and etoposide-based therapies, no difference in baseline characteristics was found except for a higher triglyceride in the former (Table 1). OS at 1 year was higher in patients who received ruxolitinib therapy although statistical significance was not met (HR 0.67; 95% CI 0.36–1.23; *p* = 0.19) [Figure 1(b)]. A subgroup analysis of patients treated between 2015 and 2023 produced similar findings (HR 0.67; 95% CI 0.42–1.07; *p* = 0.09). When the same analysis was performed between 260 anakinra and etoposide-treated patients, a significantly higher OS at 1 year was found with the former (HR 0.73; 95% CI 0.56–0.95; *p* = 0.020) [Figure 1(c)]. When stratified by treatment period, similar findings were found between 2009–2015 (HR 0.56; 95% CI 0.34–0.92; *p* = 0.021) and 2016–2024 (HR 0.57; 95% CI 0.45–0.71; *p* < 0.001). To note, baseline platelet count, hemoglobin, fibrinogen, and COVID-19 infections were higher in anakinra-treated patients while etoposide-treated patients had higher baseline alkaline phosphatase and total bilirubin (Table 1). Due to the unique immunological response elicited by the COVID-19 infection, the propensity-score matched analysis between anakinra- and etoposide-treated patients was repeated with the exclusion of those who tested positive for the COVID-19 infection. OS at 1 year remained significantly higher in anakinra-treated patients (HR 0.74; 95% CI 0.56–0.98; *p* = 0.030). A third analysis was subsequently performed between 41 patients who received ruxolitinib and anakinra and we found a similar OS at 1 year (HR 0.76; 95% CI 0.37–1.55; *p* = 0.45) [Figure 1(d)]. Analysis of patients treated between 2015 and 2023 produced nonsignificant findings (HR 1.07; 95% CI 0.65–1.76; *p* = 0.79).

**Table 1.** Baseline demographics and characteristics after propensity-score matching.

Variable^a,b^	Cohort 1		Cohort 2		Cohort 3	
	RUX (*n* = 46)	ETOP (*n* = 46)	ANAK (*n* = 260)	ETOP (*n* = 260)	RUX (*n* = 41)	ANAK (*n* = 41)
Male, no. (%)	29 (63)	32 (69.6)	134 (51.5)	138 (53.1)	23 (60.5)	24 (63.2)
Age (years)	51 ± 18	51 ± 19	41 ± 19	42 ± 19	50 ± 18	49 ± 18
Hematologic malignancy,^c^ no. (%)	31 (67.4)	28 (60.9)	70 (26.9)	65 (25)	23 (60.5)	20 (52.6)
Myeloid leukemia	13/31 (41.9)	11/28 (39.3)	NS^d^	NS^d^	NS^d^	NS^d^
Lymphoblastic leukemia	NS^d^	NS^d^	15/70 (21.4)	11/65 (16.9)	NS^d^	NS^d^
Non-Hodgkin lymphoma	11/31 (35.5)	18/28 (64.3)	37/70 (52.9)	32/65 (49.2)	NS^d^	NS^d^
NK/T-cell lymphoma	NS^d^	NS^d^	12	18	NS^d^	NS^d^
COVID-19 infection, no. (%)	NS^d^	NS^d^	**34 (13.1)**	**10 (3.8)**	NS^d^	NS^d^
Critical care services no. (%)	15 (32.6)	14 (30.4)	93 (35.8)	100 (38.5)	15 (39.5)	11 (28.9)
Baseline laboratory values						
Ferritin (mg/L)	16,864 ± 22,998	9072 ± 8978	17,082 ± 34,586	22,168 ± 44,689	18,046 ± 25,402	15,816 ± 24,912
Soluble IL-2 (pg/mL)	11,588 ± 12,104	17,137 ± 9576	11,281 ± 12,476	19,560 ± 18,193	NS^d^	NS^d^
ANC (×10^9^/L)	0.4 ± 0.4	0.5 ± 0.6	0.2 ± 1.0	0.3 ± 2.6	0.4 ± 0.4	0.5 ± 2.7
Platelet count (×10^9^/L)	96 ± 139	87 ± 123	**132 ± 130**	**93 ± 109**	102 ± 148	108 ± 129
Hemoglobin (g/dL)	8.5 ± 2.2	9.0 ± 1.8	**9.4 ± 2.3**	**8.6 ± 2.0**	8.6 ± 2.3	8.6 ± 1.7
Fibrinogen (mg/dL)	**400 ± 193**	**285 ± 194**	**337 ± 228**	**288 ± 186**	400 ± 206	318 ± 197
Lactate dehydrogenase (U/L)	950 ± 1313	1167 ± 1324	1222 ± 1623	1825 ± 1550	678 ± 652	989 ± 959
Serum creatinine (mg/dL)	1.0 ± 0.9	1.3 ± 0.9	1.4 ± 1.4	1.4 ± 1.2	1.0 ± 0.9	1.3 ± 0.9
Alkaline phosphatase (U/L)	**221 ± 216**	**383 ± 429**	**195 ± 175**	**243 ± 249**	212 ± 220	217 ± 183
Aspartate aminotransferase (U/L)	129 ± 228	317 ± 732	320 ± 770	513 ± 1337	141 ± 249	217 ± 320
Alanine transaminase (U/L)	162 ± 280	185 ± 284	204 ± 368	266 ± 608	164 ± 292	158 ± 228
Total bilirubin (mg/dL)	3.1 ± 4.7	5.6 ± 4.2	**2.6 ± 5.3**	**4.2 ± 6.9**	2.7 ± 4.4	1.5 ± 1.8
Triglyceride (mg/dL)	**225 ± 135**	**349 ± 209**	300 ± 247	320 ± 221	**213 ± 121**	**383 ± 349**

ANAK, anakinra; ANC, absolute neutrophil count; ETOP, etoposide-based therapy; ICD-10, International Classification of Diseases, Tenth Revision; IL, interleukin; NR, not reported; RUX, ruxolitinib.

a Variables that are significant with a *p* < 0.05 are bolded.

b Reported as mean ± SD unless otherwise stated.

c Indicated by composite ICD-10 code for malignant neoplasms of lymphoid, hematopoietic, and related tissue (C81–C96).

d Queries resulting in less than 10 findings are not reported by the TriNetX.

**Figure 2.**
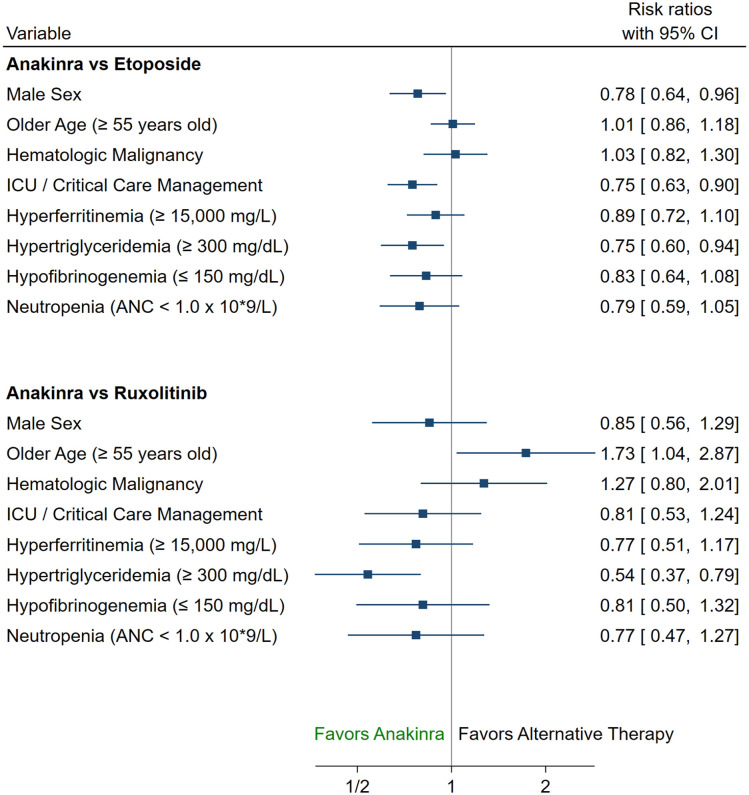
Forest plot of characteristics associated with worse 1-year OS by treatment. ANC, absolute neutrophil count; ICU, intensive care unit; OS, overall survival.

Among anakinra *versus* etoposide-treated patients, a significant survival benefit was seen with anakinra in male patients (Relative Risk [RR] 0.78; 95% CI 0.64–0.96), those who required ICU-level care for their HLH-management (RR 0.75; 95% CI 0.63–0.90), and patients with hypertriglyceridemia (RR 0.75; 95% CI 0.60–0.94). In the anakinra *versus* ruxolitinib comparison, patients who received anakinra saw survival benefits in those with hypertriglyceridemia (RR 0.54; 95% CI 0.37–0.79), although worse outcomes occurred in older patients (⩾55 years old) (RR 1.73; 95% CI 1.04–2.87) (Figure 2).

**Figure 3.**
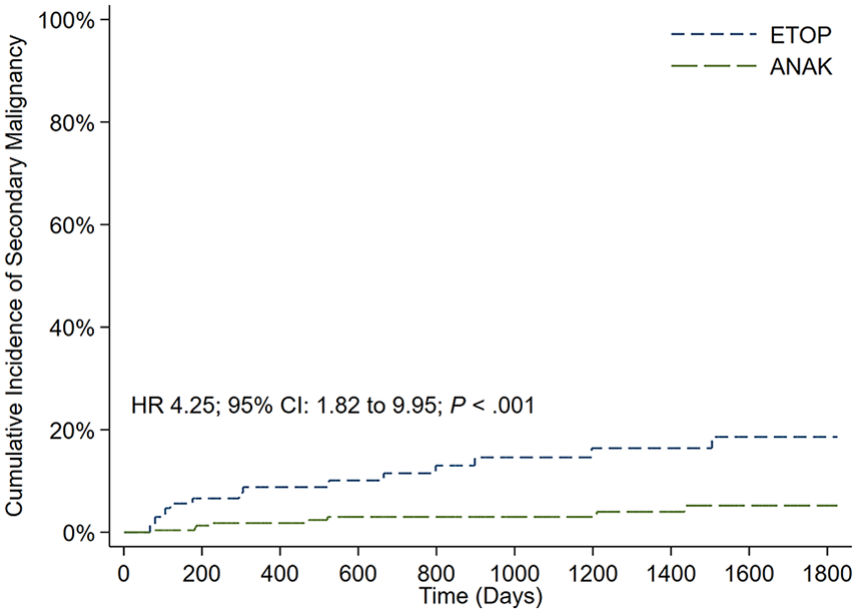
Cumulative incidence of secondary hematologic malignancy at 5 years after ETOP *versus* ANAK. ANAK, anakinra; ETOP, etoposide.

Lastly, patients who received an etoposide-based therapy had a significantly higher risk of secondary hematologic malignancy at 5 years compared to those who received anakinra (HR 4.25; 95% CI 1.82–9.95; *p* < 0.001) (Figure 3). No ruxolitinib-treated patients developed secondary malignancy at 5 years; however, this was based on a limited sample size of 15 patients.

## Discussion

The results of this large multicenter retrospective cohort study suggest that treatment with anakinra provides greater survival benefit over etoposide-based therapies for the management of adult patients with HLH. Despite long-term survival outcomes in children with HLH who received etoposide-based regimens, durability of response remains poor in adults owing largely to disease relapse and organ dysfunction.^14^ In adult patients receiving the HLH-94 protocol, 1-year OS rates are dismal ranging between 29% and 35%.^2,15,16^ In addition, the high cumulative exposure to etoposide from the HLH-94 protocol has been implicated in significantly increasing the risk of secondary hematologic malignancy.

Immune system dysregulation in patients who develop HLH involves numerous cytokines such as IL-1, IL-2, IL-6, IL-10, and interferon-gamma which are signaled through the Janus kinase (JAK) and signal transducers and activators of transcription pathways.^17,18^ Given the lack of success with etoposide-based therapies in adults, inhibition or interruption of key cytokine pathways associated with the clinical manifestations of HLH has been a clinical area of interest. Anakinra and ruxolitinib, which target IL-1 and the JAK pathway, respectively, have emerged as promising therapeutic modalities.^19,20^

Recent findings of anakinra use for HLH management have suggested therapeutic benefit in adult patients.^2–5^ Collectively, treatment response from a pooled analysis of four studies was 78.3% (*n* = 36/46) with 1-year OS reported as 33.3% (*n* = 9) and 77.8% (*n* = 6) from two studies.^2–5^ With a median follow-up of 10 months, Bavarez *et al.* also reported an OS of 88.2% in their subset of adult patients (*n* = 18) who received anakinra (one patient with prior etoposide treatment was excluded).^3^ These findings were similar to our analysis in which 1-year OS was 79.3% in the unmatched cohort. With a significantly lower rate of secondary hematologic malignancy and higher short-term survival over etoposide, our findings indicate that anakinra is a highly effective therapy for adults with HLH. Specifically, among patients who develop HLH during pregnancy or require treatment during the peripartum period, anakinra has also been demonstrated to offer a safer therapeutic option.^21^

With the first reported case of ruxolitinib use for HLH treatment in 2017,^12^ published literature investigating ruxolitinib utility is sparse, predominantly seen for relapsed/refractory disease in pediatric patients. To date, the upfront management of adults with HLH receiving ruxolitinib has been described for 46 patients, of whom 7 received monotherapy at varying doses.^6–11^ With a complete response rate of 71.4% (*n* = 5/7), ruxolitinib monotherapy appears promising albeit seen with a small sample size. Survival data are also limited to one study with case reports by Acosta *et al.*,^6^ Ahmed *et al.*,^7^ Slostad *et al.*,^8^ and Zandvakili *et al.*^9^ only describing initial response rates. Although 1-year OS was not statistically lower for ruxolitinib-treated patients in our cohort compared to etoposide-based therapies, improvement in survival was evident. In two upfront combination studies using ruxolitinib with etoposide and ruxolitinib, dexamethasone, and etoposide, with doxorubicin for adults with HLH, high response rates were seen suggesting a potential utility for initial therapy.^10,11^

This study has several limitations to acknowledge. First, although we utilized the propensity-score method to balance patient characteristics, statistically significant differences in several baseline variables were seen in the anakinra *versus* etoposide cohort. In addition, confounders such as end-organ dysfunction at the time of treatment and underlying HLH triggers were present. Second, similar to other large database studies, patient-level data are not available and factors such as treatment dosages (i.e. low-dose *versus* high-dose ruxolitinib) and underlying etiologies remain unknown. Although infectious and autoimmune triggers could not be collated due to their broad indications, we were able to balance patients with hematologic malignancies, which have been implicated as a significant source of mortality in HLH. Lastly, validation through diagnostic and prognostic tools such as the HLH-2004 criteria and H-Score could not be determined.

## Conclusion

This multicenter, retrospective electronic health cohort study indicates that anakinra is highly effective for the management of adult patients with HLH. Anakinra usage was associated with significantly lower development of secondary hematologic malignancy and higher 1-year OS when compared to etoposide-based therapies. Further studies are warranted to validate these findings.

## Declarations

**Ethics approval and consent to participate:** The study was performed using the TriNetX database which uses de-identified aggregate patient data for analysis. No protected health information or personal data are made available to the users of the platform. As a result, institutional review board approval was not required for this study.

**Consent for publication:** Not applicable.

**Availability of data and materials:** The data utilized for this study are available from TriNetX and restrictions apply to the availability of the data. Data can be made available at https://www.trinetx.com with the permission of TriNetX.
